# Biogas‐producing microbial composition of an anaerobic digester and associated bovine residues

**DOI:** 10.1002/mbo3.854

**Published:** 2019-05-25

**Authors:** Carolina Senés‐Guerrero, Franco A. Colón‐Contreras, Javier F. Reynoso‐Lobo, Benito Tinoco‐Pérez, Jorge H. Siller‐Cepeda, Adriana Pacheco

**Affiliations:** ^1^ Tecnologico de Monterrey, Escuela de Ingenieria y Ciencias Centro de Biotecnologia‐FEMSA Monterrey Mexico; ^2^ SuKarne Culiacan Mexico; ^3^ Secretaria de Agricultura y Ganaderia Culiacan Rosales Mexico

**Keywords:** anaerobic digestion, biogas, bovine residues, DGGE, metagenomics, methanogens

## Abstract

Influenced by feedstock type and microbial inoculum, different microbial groups must precisely interact for high‐quality biogas yields. As a first approach for optimization, this study aimed to identify through time the biogas‐producing microbial community in a 10‐ton dry anaerobic digester treating cattle manure by denaturing gradient gel electrophoresis (DGGE) and metagenomics. Moreover, the associated bovine residues or feedstocks (leachate, manure, oxidation lagoon water, rumen) were also characterized to determine their contribution. A diverse and dynamic community characterized by Bacteria (82%–88%) and a considerable amount of Archaea (8%–15%) presented profiles particular to each stage of biogas production. Eukaryotes (2.6%–3.6%), mainly fungi, were a minor but stable component. Proteobacteria represented 47% of the community at the start of the run but only 18% at the end, opposite to the Bacteroidetes/Chlorobi group (8% and 20%, respectively), while Firmicutes (12%–18%) and Actinobacteria (12%–32%) remained relatively constant. Methanogens of the order Methanomicrobiales represented by several species of *Methanoculleus* were abundant at the end of the run (77%) contrary to Methanosarcinales (11%) and Methanobacteriales (0.7%). Therefore, methanogenesis mainly occurred by the hydrogenotrophic pathway. Manure and oxidation lagoon water seemed to contribute key microorganisms, while rumen dominated by *Methanobrevibacter* (72%) did not proliferate in the digester. Manure particularly possessed *Methanoculleus* (24%) and uncultured methanogens identified by DGGE, whereas oxidation lagoon was exclusively abundant in *Methanolinea* (18%) and *Methanosaeta* (19%). Leachate, as the microbial inoculum from a previous run, adequately preserved the biogas‐producing community. These results could lead to higher biogas yields through bioaugmentation strategies by incorporating higher proportions or an enriched inoculum from the relevant feedstocks.

## 1 INTRODUCTION

Biogas plants are an attractive technology for sustainable generation of renewable energy. During anaerobic digestion a complex microbial community transforms organic wastes into biogas. Therefore, this practice exemplifies a sustainable solution for waste management and energy generation. The quantity and quality of the biogas, a mixture of methane, carbon dioxide and other trace gases, appears to be controlled by the type of biomass being digested and the microbial inoculum fed into the plant (Abendroth, Vilanova, Günther, Luschnig, & Porcar, 2015; Nettmann et al., 2010; Sun, Pope, Eijsink, & Schnürer, 2015; Weiland, 2010).

Traditionally, animal manure and sludge from wastewater treatment plants have been used to generate biogas (Weiland, 2010). Animal manure and slurries from cattle and swine production have been estimated as one of the largest waste streams for biogas generation (Holm‐Nielsen, Al Seadi, & Oleskowicz‐Popiel, 2009). In the European Union, it is estimated that more than 1,284 million ton/year of manure is produced by cattle, according to an average of 38.5 kg manure days^−1^ head^−1^ (Holm‐Nielsen et al., 2009). Worldwide, in year 2016 livestock, represented 1,475 million heads of cattle, which would roughly account for 21 billion ton of animal manure (FAOStat, 2016). If left untreated or inadequately managed, animal manure becomes a major environmental problem because of nutrient leaching (N, P), ammonia evaporation and pathogen contamination. In addition, livestock production is estimated to be responsible of 18% of greenhouse gas (GHG) emissions and the anthropogenic source of 9% carbon dioxide, 37% methane and 65% nitrous oxide (Steinfeld et al., 2006). However, in most countries, only a small percentage of this waste is currently processed to generate biogas. As countries commit internationally to reduce GHG and incorporate renewable sources of energy, biogas generation and its optimization gains importance. Treated manure would also generate a residual solid and liquid fraction rich in bioavailable nutrients termed the digestate, considered a valuable end product and a mode to recycle nutrients from agriculture as it is commonly used as a biofertilizer (Weiland, 2010).

The stable operation of an anaerobic digester is dictated by a dynamic equilibrium among four bacterial groups involved in the sequential digestion of the biomass from complex polymers to simpler components that are used by methanogens to generate methane. Therefore, it is crucial to elucidate the microbial community structure and function during digestion to understand and potentially optimize the process, as it can easily explain biodigester malfunctions. First, hydrolytic bacteria transform complex polymers into sugars and amino acids, followed by the process of acidogenesis and acetogenesis to generate organic acids that are transformed into acetate, H_2_ and CO_2_ for methanogenesis. Recently, it has been suggested that fungi might play an important role in the hydrolytic stage assisting bacteria in gaining access to recalcitrant plant materials (Bengelsdorf, Gerischer, Langer, Zak, & Kazda, 2012). Therefore, it is of interest to study the whole microbial community and not only the prokaryotic component. Usually, culture‐independent techniques that rely on the analysis of the 16S rRNA gene such as sequence analysis of clone libraries, fluorescence in situ hybridization, denaturing gradient gel electrophoresis (DGGE), restriction fragment length polymorphism, and 16S amplicon sequencing are used to study these complex microbial interactions (Ariesyady, Ito, & Okabe, 2007; Bengelsdorf et al., 2012; Goberna, Insam, & Franke‐Whittle, 2009; Sun et al., 2015; Tuan, Chang, Yu, & Huang, 2014; Whitford, Teather, & Forster, 2001; Zhou, Hernandez‐Sanabria, & Guan, 2010). However, these methods might present certain bias toward specific microbial groups and, since they are based on known sequences, do not cover all microbial diversity. Today, next generation sequencing allows a real‐time assessment of the whole microbial community involved in the process with applications such as metagenomics that do not rely on PCR‐targeted amplification. These data can be used to establish taxonomy, genome composition, and metabolic potential of the microorganisms in a sample. Nevertheless, as many microorganisms are still uncultured, not all microbial components of the community are identified, and bioinformatics methods and analysis platforms not always facilitate data interpretation (Menzel, Ng, & Krogh, 2016). Hence, it is still of relevance to compare results using different approaches.

In an interest to achieve higher biogas yields and improve the fertilizer value of the digestate, addition of other agricultural substrates or residues is being considered to increase the organic content of the treated biomass (Fantozzi & Buratti, 2009; Nettmann et al., 2010). Considering bovine residues, there has always been an interest in the methanogenic composition of the rumen not only to increase meat production but also as a microbial inoculum to digest plant‐material during anaerobic digestion (Fantozzi & Buratti, 2009; Zhou et al., 2010). Mexico is in the top ten producers and exporters of bovine meat in the world (SAGARPA, 2017). One of the largest meat producers in Mexico, in an effort to adopt sustainable practices, is implementing a biodigester to treat most of its wastes. Water from the oxidation lagoon of the company as well as fresh rumen and leachate from previous digester runs is incorporated into a dry mesophilic digester in order to efficiently treat animal manure. The objective of this study was to conduct a time‐lapse composition analysis of the biogas‐producing microbial community in a 10‐ton anaerobic digester and its associated bovine residues by an integrated approach that contemplates DGGE and shotgun metagenomics. By analyzing the microbial component of the different feedstocks, we pretend to establish the best substrates for biogas generation by taking into account particular microorganisms already adapted to this type of waste.

## 2 MATERIALS AND METHODS

### 2.1 Sample collection

Residual biomass from the bovine industry associated with cattle raising and meat processing was evaluated as potential feedstocks and microbial inocula for biogas generation. Samples were taken from an oxidation lagoon (OL) treating wastewater from the meat production plant, leachate (L) from a previous biodigester run, 6‐month‐old dried cattle manure (M) and fresh rumen (R) fluid. In addition, a pilot‐scale dry anaerobic digester fed with these feedstocks and other supporting substrates (64.2% manure, 18.2% oxidation lagoon water, 3% rumen, 9.6% wood chips, 4.0% corn stover, 0.9% dust mill) was evaluated by sampling through time the recirculating leachate between two 10‐ton serial reactors. The digester was operated under batch mesophilic dry condition (60% total solids and 32% volatile solids). Biodigester (B) samples included seven points during the 22 days of operation in the following dates: 17.01.2014 (B17), 20.01.2014 (B20), 22.01.2014 (B22), 27.01.2014 (B27), 29.01.2014 (B29), 31.01.2014 (B31), and 04.02.2014 (B04). Sterile 1 L‐Nalgene bottles filled to the top were used for liquid samples (OL, L, B), while solid samples (M, R) were packed in sterile 18 oz. Whirl‐Pak bags. All samples were kept at 4°C until DNA extraction. Feedstock samples were processed during the following days (4–7 days) after arrival at the laboratory and samples from the biodigester run were processed all together after the run concluded (22 days). A preliminary evaluation of sample processing time was conducted by comparing DGGE profiles (data not shown), the same community profiles were obtained from samples stored for 4–7 days compared to around one month.

### 2.2 DNA extraction

Different volumes of the liquid samples (25 and 50 ml) were first evaluated to optimize extraction. Samples were filtered through a series of pore sizes to remove large particles (coffee filter, 20–25 μm and 2.5 μm) until a cell pellet was obtained at the last filtration step (0.45 or 0.22 μm). By comparing DGGE profiles (data not shown), it was established that 25 ml filtered to 0.45 μm was ideal to reduce sample manipulation in a timely matter. Filters from liquid samples and 0.5 g from solid samples were used for DNA extraction using the FastDNA Spin Kit for Soil (MP Biomedicals, USA). DNA quality was evaluated by agarose gel electrophoresis and spectrophotometry (Nanodrop 1000, Thermo Scientific, USA), and quantified by fluorometry (Qubit 2.0, Invitrogen, USA).

### 2.3 Denaturing gradient gel electrophoresis analysis

A nested PCR approach was used for targeted amplification of methanogens as described by Zhou et al. (2010). First, a large 800 bp fragment of the 16S rRNA gene was amplified (94°C, 5 min; 30 cycles of: 94°C for 30 s, 57°C for 30 s, 68°C for 60 s; and 68°C, 7 min) with primers Met86f (5‐GCTCAGTAACACGTGG‐3) and Met915r (5‐GTGCTCCCCCGCCAATTCCT‐3). Then a nested PCR (1:100 dilution of previous PCR product) was performed with primers GC‐ARC344f (5‐ACGGGGYGCAGCAGGCGCGA‐3) and 519r (5‐GWATTACCGCGGCKGCTG‐3), the forward primer possessed a 40 bp GC‐clamp, which targeted a 191 bp fragment in the 16S rRNA‐V3 region (95°C, 5 min; 30 cycles of: 95°C for 30 s, 56.5°C for 30 s, 72°C for 30 s; and 72°C, 7 min). PCR products were loaded on a 1% (w/v) agarose gel with 1X Tris‐acetate‐EDTA (TAE) buffer and visualized after ethidium bromide (0.5 g/L) staining.

Denaturing gradient gel electrophoresis of PCR products was performed using a DCode Universal Mutation System (Bio‐Rad, USA) in 1X TAE buffer with a 1.0 mm‐thick vertical gel containing 6% (w/v) polyacrylamide (37.5:1 acrylamide:bisacrylamide) and a 35–55% (w/v) linear gradient of denaturants (100% denaturation solution contained 7 M urea and 40% (w/v) formamide). Gel wells were loaded with 35–45 µl of the nested PCR product according to agarose gel band intensity and 1⁄4‐volume of loading buffer. Running conditions were 3.5 hr at 150 V. After, the gel was stained with ethidium bromide according to the manufacture's protocol, visualized on a UV transilluminator at 312 nm using a Molecular Imager ChemiDoc XRS System (Bio‐Rad, USA). The most intense bands were excised in the middle with RNase/DNase clean scalpels and DNA was eluted according to Chory and Pollard (2001). An aliquot (2 µl) was used for PCR re‐amplification using conditions described above and a second DGGE was run to confirm band purity. PCR products were cleaned with a PCR Clean‐Up System (Promega, USA) and sequenced with the same primer pair without the GC clamp at Eton Bioscience, Inc. (San Diego, USA). To determine the closest known relative species, sequences were blasted against the NCBI GenBank and MiDAS 2.1 database (Mcllroy et al., 2017). Sequences were deposited in NCBI under accession numbers MH393448‐MH393458.

Similarities among DGGE community profiles were defined by analyzing gel images using ImageJ 1.48 (Rasband, 1997). Bands of each lane were detected automatically and their relative intensity measured by the peak area. Bands with <1% intensity with respect to the total intensity of the lane were removed from the analysis. A Manhattan distance matrix was generated for pairwise comparisons between lanes using MeV_4_8 v. 10.2 (Saeed et al., 2003). This matrix was used for hierarchical clustering using the unweighted pair group method with arithmetic mean.

### 2.4 Shotgun metagenomic analysis

Genomic DNA was used for library preparation using Nextera XT DNA Library Prep Kit (Illumina, USA) according to the manufacturer's protocol. Libraries were sequenced (151 bp paired‐end) with MiSeq Reagent Kit v3, 600 cycles, using the MiSeq system (Illumina, USA) at the sequencing facilities of Tecnologico de Monterrey (Monterrey, Mexico). Raw sequenced data were processed by the FASTQ Toolkit v2.2.0 (BaseSpace Labs, Illumina, USA) to trim adapter sequences and remove reads below a mean quality of Q20, unpaired reads and reads <32 bp length. Taxonomic characterization was done by the metagenomic classifier Kaiju (Menzel et al., 2016) using the NCBI BLAST *nr* + euk reference dataset, searching for maximum exact matches with a minimum match length of 11. Kaiju classifies individual metagenomic reads using a reference database comprising the annotated protein‐coding genes of a set of microbial genomes. For this study, the reference dataset contained 89 M protein sequences from Bacteria, Archaea, virus, Fungi, and other microbial eukaryotes. All sequences have been archived in the NCBI Sequence Read Archive under BioProject no. PRJNA378243. Relative read abundance as the proportion of raw reads of each taxon from the total amount of reads was used to assess the distribution of taxa across the different samples. Reads that were not assigned to any taxa at the phylum level were removed from the analysis.

For multivariate statistical analysis of metagenomic data, raw reads were normalized using DESeq2 (Love, Huber, & Anders, 2014) with the counts function and the parameter normalized=TRUE. A normalized data matrix of phyla from each microbial domain was used for hierarchical clustering using the package Pvclust (Suzuki & Shimodaira, 2006) with Ward's method and a bootstrap number of 10,000. This data matrix was also analyzed by principal component analysis (PCA) with Euclidean distances and the total amount of inertia equal to number of species, using the vegan package (Oksanen et al., 2017). All statistical analyses were conducted in R v.3.3.2 (R Core Team, 2016).

## 3 RESULTS

### 3.1 Performance of the 10‐ton anaerobic digester

**Figure 1:**
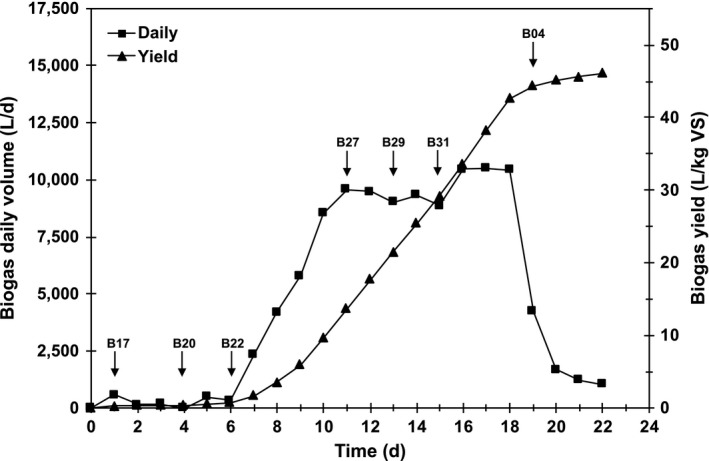
Biogas production from anaerobic digestion of bovine residues in a 10‐ton pilot biogas plant under mesophilic dry fermentation conditions. Arrows indicate sampling time for microbial analysis

During sampling, the biodigester showed a lag‐phase of 6 days followed by biogas formation and 8 days of peak production until activity came to a halt (Figure 1). Residual oxygen in the biodigester decreased from day 1 to 8 from 3.8% to 2.1% v/v and remained around 2% during biogas production. Volatile fatty acids (VFAs) in the digester leachate increased from 2.4 ± 0.2 g acetic acid L^−1^, at the start of the run, to 14.4 ± 0.7 g/L at peak gas production and significantly decreased to a range of 4.6–7.6 g/L by the end of the run, indicating biomass breakdown and conversion of VFAs. Other performance parameters during the run showed a pH of 6.8–7.9, total inorganic carbon (TIC) of 5.6–9.3 g CaCO_3_ L^−1^ and VFAs/TIC ratios of 0.4–1.34. At peak production, 9.7 ± 0.7 m^3^ days^−1^ of biogas were produced with 49.6 ± 3.3% methane content. After 22 days of digestion, biogas and methane yield (46.1 L biogas kg VS^−1^ and 25.5 L CH_4_ kg VS^−1^, 55.3% CH_4_) were in the low range of reported values for mesophilic treatment of cattle manure due to mechanical problems with the run (i.e. pump failure during leachate recirculation at day 4 to day 14, when it was re‐establish). Although, biogas production peak during this period with an accumulation of VFAs that were later consumed. Fantozzi and Buratti (2009) reported productivities of 40 L CH_4_ kg VS^−1^ for bovine fresh manure in a laboratory reactor (17 L working volume) and cited literature values of 170 to 220 L CH_4_ kg VS^−1^. However, these reports contemplate thermophilic operations and not necessarily dry fermentation conditions.

### 3.2 Methanogen DGGE analysis

**Figure 2:**
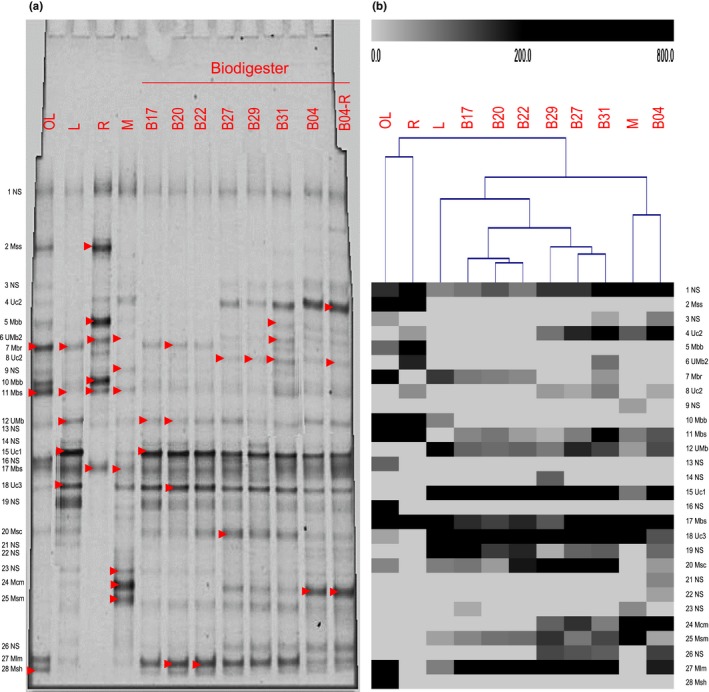
Denaturing gradient gel electrophoresis gel of archaeal PCR products (a) and band pattern analysis (b) showing hierarchical clustering of samples and band intensity. Arrows indicate cut bands from gel. Samples: OL, oxidation lagoon; L, leachate; R, rumen; M, manure; B, biodigester time series (B17 = 17.01.2014, B20 = 20.01.2014, B22 = 22.01.2014, B27 = 27.01.2014, B29 = 29.01.2014, B31 = 31.01.2014, B04 = 04.02.2014, B04‐R = 04.02.2014‐replicate). Band identity: NS, not sequenced; Mss, *Methanosphaera stadtmanae*; Uc, uncultured archaeon; Mbb, *Methanobrevibacter boviskoreani*; Mbs, *Methanobrevibacter smithii*; Mbr, *Methanobrevibacter ruminantium*; UMb, uncultured *Methanobrevibacter*; Msc, *Methanosaeta concilii*; Mcm, *Methanoculleus marisnigri*; Msm, *Methanosarcina mazei*; Mlm, *Methanolinea mesophila*; Msh, *Methanospirillum hungatei*

Our first approach contemplated DGGE analysis of archaeal PCR products from the V3 region of the 16S rRNA gene. In this technique, community profiles from different samples were evaluated by studying the most abundant members, which corresponded to the most intense bands in the DGGE profile (Figure 2a). A total of 38 bands were cut from the gel and 68% of bands were successfully purified and sequenced. These bands were annotated based on their closest similarity to cultured or uncultured species in two gene databases. Nine methanogen species were identified, and five bands were classified as uncultured archaeons (Table S1). Most sequences belonged to the *Methanobrevibacter* genus, which seemed to produce a multiple band pattern. Different strains of *Methanobrevibacter smithii* (Mbs) were present in almost all samples (Figure 2a, Band 6, 11, 17) and *Methanobrevibacter ruminantium* (Mbr) dominated the oxidation lagoon (OL), while *Methanobrevibacter boviskoreani* the rumen (R) (Figure 2a, Band 7, 10). Even though they were not as ubiquitous, intense bands of uncultured archaeon 1 (Uc1) and 3 (Uc3) (Figure 2a, Band 15, 18), and *Methanolinea mesophila* (Mlm; Figure 2a, Band 27) characterized the biodigester run (B17‐B04). Leachate (L) also presented these bands and the oxidation lagoon (OL) was the only other feedstock where *M. mesophila* was observed. At the end of the biodigester run, uncultured archaeon 2 (Uc2) and *Methanoculleus marisnigri* (Mcm) became abundant (Figure 2a, Band 4, 24). The latter also dominated the manure (M) sample. Overall, methanogen richness (14–17 bands; Figure 2a) was the highest in the biodigester after 10 days into the run when maximum biogas production rates were observed (Figure 1). Among feedstocks, oxidation lagoon (OL) was the most diverse (14 bands) and rumen (R) the least (9 bands) (Figure 2a). By comparing the gel band pattern of each community, it was determined that the first days of the biodigester run (B17‐B22, Figure 2b) were more similar among each other than the middle (B27‐B31) and last sampled day (B04). All biodigester samples shared a high similarity to leachate (L), with the exception of the last day (B04) that was more similar to manure (M) (Figure 2b). Feedstocks oxidation lagoon (OL) and rumen (R) shared similar methanogen compositions and differed from the biodigester run and other feedstocks.

**Table 1:** Number of paired‐end reads and read relative abundance in parenthesis (%) for each microbial domain (based on classified reads)

Domain	L^a^	M	OL	R	Biodigester				
					B17	B22	B27	B29	B04
Eukaryota	14,726 (2.5)	13,378 (1.2)	8,081 (1.5)	22,984 (1.9)	9,288 (3.1)	16,575 (3.1)	19,823 (3.6)	27,846 (2.8)	17,706 (2.6)
Bacteria	472,618 (79.9)	1,083,849 (97.9)	504,931 (95.9)	1,187,699 (96.1)	255,101 (84.5)	440,644 (82.1)	487,003 (88.1)	858,988 (86.6)	594,111 (88.2)
Archaea	102,535 (17.3)	8,123 (0.7)	12,674 (2.4)	22,692 (1.8)	36,553 (12.1)	77,900 (14.5)	44,203 (8.0)	102,580 (10.3)	59,013 (8.8)
Virus	2,019 (0.3)	1,960 (0.2)	893 (0.2)	2,571 (0.2)	1,074 (0.4)	1,438 (0.3)	1,922 (0.4)	2,912 (0.3)	2,797 (0.4)
Classified reads	591,898 (42)^b^	1,107,310 (61)	526,579 (49)	1,235,946 (45)	302,016 (47)	536,556 (52)	552,951 (42)	992,326 (44)	673,627 (44)
Total paired‐end reads	1,417,913	1,809,324	1,074,462	2,762,894	643,912	1,039,131	1,328,532	2,266,266	1,544,364

a Samples: L, leachate; M, manure; OL, oxidation lagoon; R, rumen; B, biodigester time series (B17 = 17.01.2014, B22 = 22.01.2014, B27 = 27.01.2014, B29 = 29.01.2014, B04 = 04.02.2014).

b Percentage of classified reads out of the total amount of paired‐end reads passing quality filters.

### 3.3 Shotgun metagenomic analysis

#### 3.3.1 Whole‐microbial community at high taxonomic level

To analyze all microbial components of the samples a metagenomic approach was performed. Sequencing of all sample libraries resulted in 27,773,646 single reads passing quality filters, which corresponded to 97.9% total reads (individual reads per sample are shown in Table S2). Taxonomic identity per sample was possible in 42%–61% of reads (Table 1). The manure (M) feedstock showed the highest percentage of classified reads (61%), while leachate (L) along with the last days of the biodigester run presented the least classified (56%–58%). Table 1 shows that the major microbial domain in all samples was Bacteria with a relative abundance of 79.9%–97.9%, followed by Archaea (0.7%–17.3%), Eukaryota (1.2%–3.6%) and a small not that variable percentage of virus (0.2%–0.4%). All biodigester run samples presented higher amounts of Archaea (8.0%–14.5%) compared to feedstocks (0.7%–2.4%), except for leachate (L) with a relative abundance of 17.3%. Also, the Eukaryota component was higher in the biodigester (2.6%–3.6%) compared to feedstocks (1.2%–2.5%).

**Figure 3:**
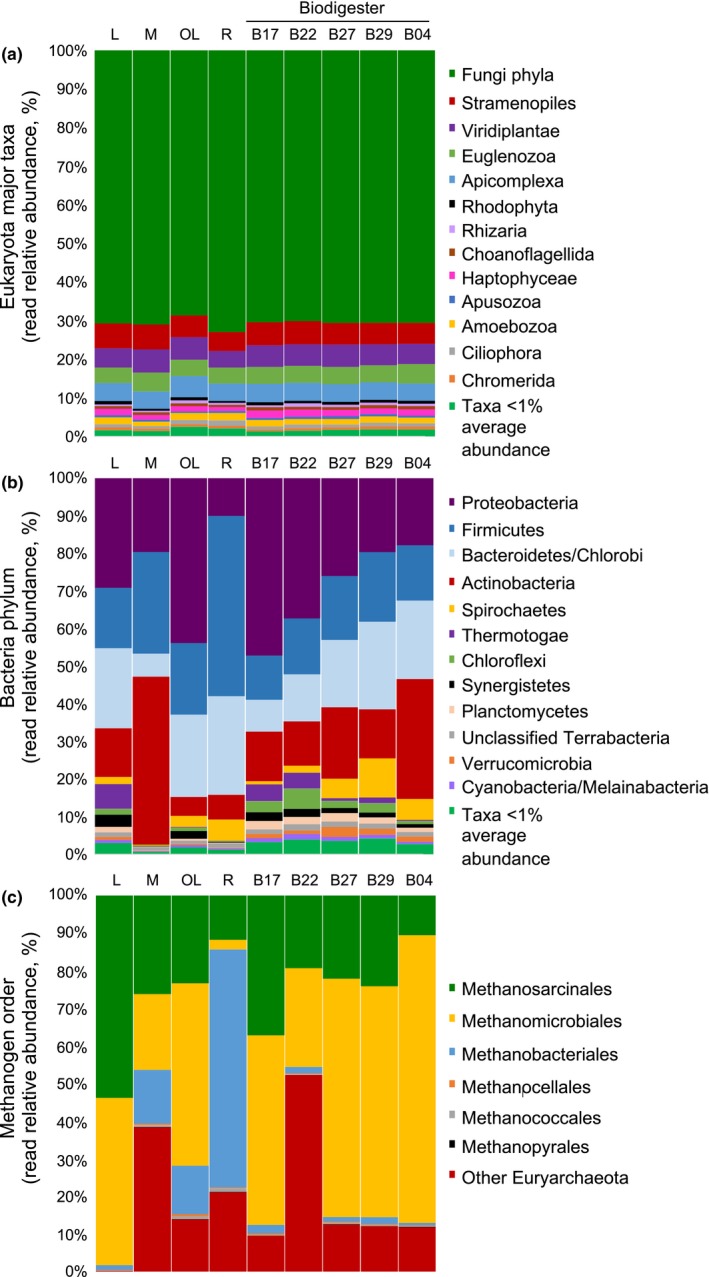
Read relative abundance of Eukaryota major taxa (a), bacterial phyla (b), and methanogen orders (c). Samples: L, leachate; M, manure; OL, oxidation lagoon; R, rumen; B, biodigester time series (B17 = 17.01.2014, B22 = 22.01.2014, B27 = 27.01.2014, B29 = 29.01.2014, B04 = 04.02.2014)

As community dynamics of eukaryotes and bacteria influence the ability of methanogens to generate biogas, classified reads were further analyzed at lower taxonomic levels (Figure 3). All samples enclosed a similar composition of major eukaryote groups, where fungi predominated (69%–73%) (Figure 3a). The Bacteria domain was led by four phyla (Proteobacteria, Firmicutes, Bacteroidetes/Chlorobi and Actinobacteria) that differed in read relative abundance among samples. In the biodigester samples, differences in the Bacteria component seemed to response to time. However, as microbial biomass was not assessed per sample, direct comparisons among samples can be misleading. Proteobacteria at day B17 represented 47% of the community but by day B04 only 18%. On the contrary, the proportion of the Bacteroidetes/Chlorobi group was 8% at the start of the run and at the end was around 20%, similar to Actinobacteria (Figure 3b). Firmicutes proportions in all biodigester samples were between 12%–18% and Spirochaetes seemed to be important components of the community at the end of the run (6%–10%). Among feedstocks, bacteria composition was distinct for each sample. Leachate (L) was the most uniform community, resembling the biodigester run where Proteobacteria and Bacteroidetes/Chlorobi were the main phyla with an abundance each of 25%–30% followed by Firmicutes and Actinobacteria (Figure 3b). Actinobacteria dominated in manure (M) with an abundance of 44.7%, whereas Proteobacteria accounted for 43.8% of reads in the oxidation lagoon (OL). Finally, the rumen (R) consisted mostly of Firmicutes (47.9%).

The Archaea community in all samples was predominantly of the phylum Euryarchaeota (89.5%–97.5%) with 1.4%–4.5% unclassified Archaea (data not shown). Interestingly, the manure sample also presented a proportion of Crenarchaeota (5.1%) and Thaumarchaeota (2.0%). Lower abundances of Crenarchaeota were also found in the oxidation lagoon and rumen (2.4%–3.0%). Methanogens, which belong to the phylum Euryarchaeota, were represented by the orders Methanosarcinales, Methanomicrobiales and Methanobacteriales (Figure 3c). In addition, there was a significant contribution of reads classified as other Euryarchaeota (0.1%–52.3%). During the biodigester run, Methanomicrobiales was the most abundant order (26%–77%) followed by Methanosarcinales (11%–37%) and a low representation of Methanobacteriales (0.7%–2.3%). However, at day B22 there was a shift toward other Euryarchaeota but the community reestablished by day B27, when biogas was steadily produced (B27 and B29, Figure 1). Therefore, peak biogas production was characterized by the dominance of the order Methanomicrobiales (61%–64%), a slight reduction in Methanosarcinales (22%–24%) and the reestablishment of lower levels of other Euryarchaeota (12%–13%). Among feedstocks, leachate (L) was almost equally represented by Methanomicrobiales and Methanosarcinales, and the oxidation lagoon (OL) presented a similar profile as the biodigester but with a higher proportion of Methanobacteriales. Lastly, Methanobacteriales dominated the rumen (R) community (63.3%) and was also present in manure (M), along with Methanomicrobiales, Methanosarcinales and an important contribution of other Euryarchaeota.

**Figure 4:**
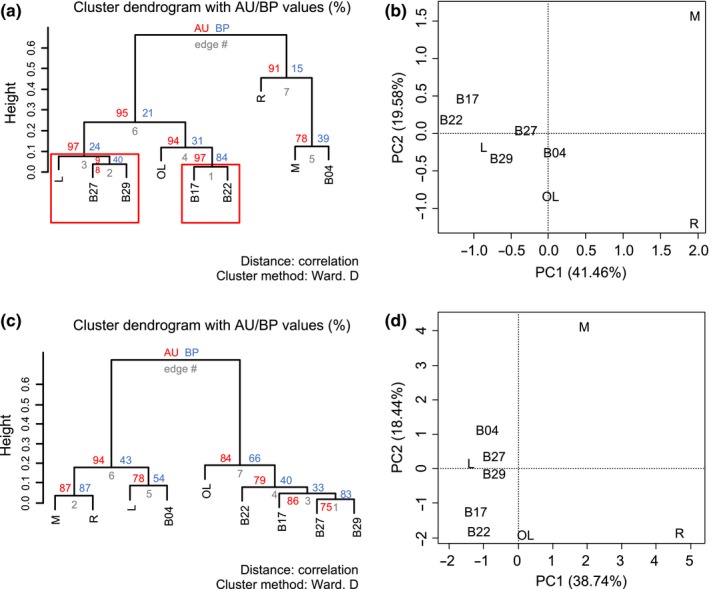
Hierarchical clustering and principal component analysis of the metagenomes from samples annotated at the phylum level (a–b) and of methanogens at the order level (c–d). Red squares represent clusters with >95% AU supported *P*‐values. AU, approximately unbiased; BP, bootstrap probability; Samples: L, leachate; M, manure; OL, oxidation lagoon; R, rumen; B, biodigester time series (B17 = 17.01.2014, B22 = 22.01.2014, B27 = 27.01.2014, B29 = 29.01.2014, B04 = 04.02.2014)

Similarities among the metagenome of each sample, considering Eukaryota, Bacteria and Archaea, were evaluated by hierarchical clustering (Figure 4). At the highest taxon level, significant (*p* ≤ 0.05) microbial community profile similarities were found between leachate (L) and the middle days of the biodigester run (B27, B29), while a second cluster corresponded to the first days of the biodigester (B17, B22) (Figure 4a). PCA supported these groupings but showed that the last day of the biodigester run (B04) was more similar to oxidation lagoon (OL) than manure (M) (Figure 4b). Moreover, manure (M) and rumen (R) did not group with the biodigester samples. Although not statistically significant, methanogen community profiles at the order level clustered similarly to the whole community analysis, with the exception of leachate (L) which grouped with the branch comprised of the last day of the biodigester run (B04), manure (M) and rumen (R) (Figure 4c). Methanogen PCA grouped all biodigester samples with leachate (L), while oxidation lagoon (OL), manure (M), and rumen (R) each formed an independent group (Figure 4d).

#### 3.3.2 Methanogen community at genus and species level

**Figure 5:**
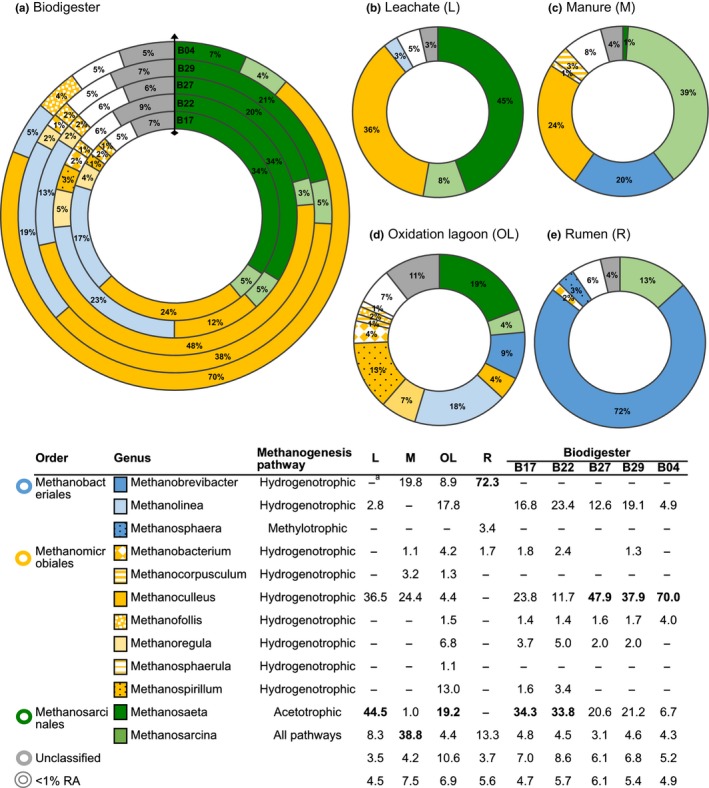
Distribution of methanogens at the genus level (≥1% relative abundance) in the biodigester run (a) and associated feedstocks (b–e). Proportions of the genus relative abundance are shown in the table. ^a^Relative abundance <1%, considered in category <1% RA. Samples: L, leachate; M, manure; OL, oxidation lagoon; R, rumen; B, biodigester time series (B17 = 17.01.2014, B22 = 22.01.2014, B27 = 27.01.2014, B29 = 29.01.2014, B04 = 04.02.2014)

Overall, methanogen orders were represented by 12 genera and 33 species, which possessed ≥1% read relative abundance in at least one sample (Figure 5 and Table S3). Hydrogenotrophic and acetotrophic methanogens were found in high abundances, contrary to methylotrophs only represented by the genus *Methanosphaera* in the rumen (R) sample (Figure 5). In the biodigester run, the most abundant orders (Methanomicrobiales, Methanosarcinales, and Methanobacteriales) were represented each by a genus (*Methanoculleus*, *Methanosaeta*, and *Methanolinea*, respectively) that seemed to change in abundance with time. *Methanoculleus*, a hydrogenotroph, dominated the biodigester community (70% relative abundance) by the end of the run, while *Methanosaeta,* an acetotroph, and *Methanolinea,* a hydrogenotroph, were minor components (7% and 5%, respectively) (Figure 5a). At peak biogas production, these genera presented intermediate abundances in the observed range under the dominance of *Methanoculleus* (48%–38%). Other minor members were *Methanoregula* and *Methanosarcina* (2%–5%). All other genera showed lower abundances (<4%) and were present at specific sampling times, *Methanospirillium* and *Methanobacterium* during the first days, while *Methanofollis* increased at the end of the run. The proportion of genera below <1% read relative abundance (5%–6%) and unclassified methanogens (5%–9%) remained constant throughout the run.

The feedstocks leachate (L), manure (M) and rumen (R) (Figure 5b,c,e) were dominated each by a different methanogen, *Methanosaeta* (45%), *Methanosarcina* (39%) and *Methanobrevibacter* (72%), respectively. Leachate (L) and manure (M) also presented a high proportion of *Methanoculleus* (36 and 24%, respectively). On the contrary, oxidation lagoon (OL) presented a diverse methanogen community (Figure 5d), where *Methanosaeta* (19%) and *Methanolinea* (18%) were the most abundant genera. Interestingly, *Methanobrevibacter* was not observed in the biodigester even though it was an abundant component of rumen (72%) and manure (20%).

Relevant methanogen species in the biodigester run were consistent with the pattern described for genera. *Methanoculleus*, as one of the most abundant genera, was represented by eight species (Table S3), where *M. marisnigri* was the most abundant at the end of the run (7%) followed by *M. horonobensis* and strain MH98A (6%). On the contrary, *Methanosaeta*, represented by *Methanosaeta concilii* and *Methanosaeta harundinacea*, were abundant at the start of the run (11%–13%). At peak biogas production the most abundant species in decreasing order were *M. concilii* (15%–17%), *Methanolinea* sp. strain SDB (8%–14%), *Methanolinea tarda* (4%–5%) and *M. marisnigri* (5%–4%). However, as mentioned above, *Methanoculleus* was represented by several species that add up to 20%–25% read relative abundance followed by *Methanosaeta* (18%–19%) and *Methanolinea* (12%–19%) species. Other species that appeared at the end of the run (B04) but in low abundance (around 2%) were *Methanofollis ethanolicus*, *Methanofollis liminatans* and *Methanosarcina mazei*. Interestingly, the most abundant species in the feedstock leachate (L), *M. harundinacea* (20%), was not the species of *Methanosaeta* that proliferated in the biodigester during peak production. Instead, *M. concilii* was the main species, probably contributed by the oxidation lagoon (OL) where it was the major component (18%). In addition, leachate (L) together with manure (M) presented most of the species of the genus *Methanoculleus* that were observed in the biodigester. Still, manure (M) was dominated by *M. mazei* (11%), which was not relevant in the digester. Similarly, most rumen (R) species also represented by an abundance of *M. mazei* (12%) and 10 species of *Methanobrevibacter* (9% *M. ruminantium*, 6% *M. olleyae*, 6% *Methanobrevibacter millerae*) were not observed in the biodigester. On the contrary, the oxidation lagoon (OL) presented most of the species during peak biogas production (18% *M. concilii*, 11% *M. tarda*, 6% *Methanolinea* sp. SDB) except for *Methanospirillum hungatei* (13%), which was in low abundance (2%–3%) only at the beginning of the run. Nevertheless, species‐level interpretation should be cautious due to the high amount of sequences (23%–47%) that could not be classified to known species (Table S3).

## 4 DISCUSSION

Methanogens play a key role in biogas production and, therefore, have been the focus of many microbial community studies (Guo et al., 2015; Nettmann et al., 2010; Traversi, Villa, Lorenzi, Degan, & Gilli, 2012; Whitford et al., 2001; Zhou et al., 2010). Current high‐throughput sequencing technologies allow a deeper insight into the whole microbial community structure and functioning, shifting the attention on understanding complex interactions to optimize biogas yield (Guo et al., 2015; Stolze et al., 2015; Sun et al., 2015; Wirth et al., 2012; Yang et al., 2014). In this study, two approaches were used to unravel the microbial community structure of a 10‐ton anaerobic digester designed to treat bovine residues under mesophilic dry fermentation conditions. DGGE was used to target methanogens and a metagenomic approach was used to study the whole microbial community. As expected, DGGE failed to cover and resolve many species that were assessed by the metagenomic study. Multiple DGGE bands were assigned to the same methanogen species and some incongruities between techniques were detected probably due to DGGE identity assignment by band positioning (Figure 2). For example, the genus *Methanobrevibacter*, which characterized the most abundant members of the feedstock rumen (R), was represented by three species of which *M. smithii* and *M. boviskoreani* showed multiple DGGE band patterns (Figure 2, Band 5–7, 10, 11, 17). Conversely, the metagenomic analysis identified ten *Methanobrevibacter* species and strains suggesting that some of these bands might correspond to different genotypes, underestimating this genus diversity (Table S3). Zhou et al. (2010) found similar DGGE patterns for several methanogen genera. For example, five bands appeared to correspond to different strain sequence types of *Methanobrevibacter gottschalkii*. Other unrepresented member in the DGGE analysis was the genus *Methanoculleus* with only one species identified (Figure 2, Band 24), while the metagenomic study recovered eight abundant species and strains (Table S3). Nonetheless, we were able to elucidate with both methods the most abundant methanogen genera that characterized each microbial community. Also, both approaches were consistent in the role that uncultured or unclassified methanogens might play during anaerobic digestion. DGGE bands classified as uncultured methanogens were among the most intense bands during the biodigester run (Figure 2, Band 4, 15, 18). Accordingly, the metagenome study showed a high proportion of the reads (23%–48%) that corresponded to unclassified methanogens at the species level (Table S3). Thus, the DGGE analysis was relevant to qualitatively identify changes in the microbial community related to the uncultured component of the community.

Understanding how to maintain a balance among the four microbial metabolic stages of biogas production (hydrolysis, acidogenesis, acetogenesis, and methanogenesis) is key to improve productivity (Weiland, 2010). However, the microbial process of generating biogas cannot be generalized as it has been shown that microbial diversity and shifts in the community strongly depend on the type of substrate being treated and the reactor system (Abendroth et al., 2015; Bengelsdorf et al., 2012; Nettmann et al., 2010; Weiland, 2010). Nevertheless, extremely stable bacterial and methanogenic community profiles have also been reported (Goberna et al., 2009; Kampmann et al., 2012; Stolze et al., 2015), generally associated with higher taxonomic levels and attributed to functional redundancy among phylogenetic groups or being defined by a crucial process parameter such as high salt content (Goberna et al., 2009).

In this study, fungi were dominant players among the eukaryote microbial community (Figure 3a). Surprisingly, their abundance was almost the same in every sample but during biogas production represented a higher percentage of the community, which might suggest a key role during biogas generation (Table 1). Stable fungal presence in biogas plants has been reported (Bengelsdorf et al., 2012), but knowledge of their role remains unclear. It has been suggested that fungi assist in lignocellulose decomposition, penetrating the lignified material first for cellulolytic bacteria to gain access. Contrary to what was observed with Eukaryota, Bacteria composition considerably varied during biogas generation and among feedstocks (Figure 3b). It has been shown that for treatment of solid feedstocks, the community is usually dominated by Firmicutes (Abendroth et al., 2015; Stolze et al., 2015; Tuan et al., 2014; Wirth et al., 2012). Our results showed that Firmicutes only dominated the rumen (R) feedstock and, during biogas production, Firmicutes was third in frequency and characterized by a stable presence throughout time. Kampmann et al. (2012) also reported Firmicutes as a stable phylum during liquid manure treatment. Other studies associated with the digestion of liquid feedstocks as sludge have reported Proteobacteria, Firmicutes and Bacteroidetes as dominant bacterial phyla, followed by Actinobacteria and Chloroflexi (Guo et al., 2015; Yang et al., 2014). Spirochaetes has also been observed as an abundant phylum along with Bacteroidetes when treating sludge (Abendroth et al., 2015). These six phyla collectively characterized the biodigester community that changed in abundance during the run. Each feedstock seemed to contribute a different bacterial group to the biodigester as a particular phylum dominated each residue: oxidation lagoon (OL) by Proteobacteria, manure (M) by Actinobacteria and rumen (R) by Firmicutes. During peak biogas production, Proteobacteria decreased in abundance opposite to Bacteroidetes/Chlorobi, while Firmicutes and Actinobacteria remained stable and Spirochaetes, a minor component, also increased (Figure 3b). It seems that hydrolytic bacteria were present and active from the start of the run, first represented by members of the phyla Firmicutes and Actinobacteria, and then assisted by an increase of the Bacteroidetes/Chlorobi group. Firmicutes and Bacteroidetes members possess cellulose and hydrogenase activity (Wirth et al., 2012), while Actinobacteria produce lignin‐degrading enzymes that break down complex organic materials (Wirth et al., 2012; Yang et al., 2014). In addition, Firmicutes and Bacteroidetes participate in the fermentation of the generated products into organic acids, CO_2_ and H_2_ (Traversi et al., 2012; Wirth et al., 2012). Also, Firmicutes can proceed with the consumption of butyrate and various VFAs (Ariesyady et al., 2007). These hydrolytic bacteria seemed to be assisted in glucose degradation by Spirochaetes and Proteobacteria known to consume glucose, propionate, butyrate, and acetate. The observed decrease of Proteobacteria in the biodigester, probably incorporated by the use of water from the oxidation lagoon, might be related to the capacity of other feedstocks to contribute microbial species already adapted to the treated substrates in the digester as rumen and manure, which were abundant in Firmicutes and Actinobacteria, respectively. Abundance of Chloroflexi and Proteobacteria has been correlated to low biogas yield while Firmicutes and Bacteroidetes characterized high biogas production (Abendroth et al., 2015). In this study, the latter phyla were observed to remain stable or even increase in abundance during biogas peak production while the former decreased.

Methanogenesis as the last crucial step in anaerobic digestion is where the stability of the process is more susceptible (Traversi et al., 2012). Our results showed a high representation of Archaea in the microbial community associated to the biodigester and leachate (8%–17%, Table 1). Other metagenomic studies of biogas plants have reported 6%–10% archaeal abundance (Guo et al., 2015; Stolze et al., 2015; Wirth et al., 2012). Methanogens were represented by the orders Methanosarcinales, Methanomicrobiales and Methanobacteriales (Figure 3c) and their abundance varied accordingly to feedstock and the biodigester time course, as observed with bacteria. In the biodigester, the order Methanomicrobiales dominated, and was represented by several species of the genus *Methanoculleus* with the most abundant species being *M. marisnigri* followed by *M. horonobensis* (Figure 5, Table S3). *Methanoculleus* is frequently described as the dominant Archaea in biogas plants, particularly in the treatment of solid feedstocks and in reactors with inorganic supports or high total solid content (Abendroth et al., 2015; Goberna et al., 2009; Nettmann et al., 2010; Stolze et al., 2015; Weiland, 2010; Wirth et al., 2012; Zhao et al., 2013). The presence of this methanogen has also been correlated to critical process parameters such as high concentrations of ammonia and salt (Goberna et al., 2009; Nettmann et al., 2010). *Methanoculleus* is capable of forming biofilms increasing its capability to attach to solids and tolerate inhibitor substances and reactor disturbances (Abendroth et al., 2015; Goberna et al., 2009). This characteristic might explain the increase in abundance of this genus with time and entire dominance by the end of the biodigester run (Figure 5). As methanogenesis pathways are well known for methanogen genera, it can be suggested that the main pathway in the biodigester was the hydrogenotrophic, as *Methanoculleus* is known to use H_2_ and CO_2_ to generate methane (Anderson et al., 2009). However, the second most abundant order Methanosarciales mainly represented by *M. concilii* uses the acetotrophic route for methanogenesis. Conversely, because the genus *Methanoculleus* was represented by several relevant species and the third most abundant order Methanobacteriales is also a hydrogenotroph, this pathway dominates the studied biogas reactor. *Methanosaeta* is favored under low acetate conditions commonly found in sludge digesters (Abendroth et al., 2015; Guo et al., 2015; Yang et al., 2014). In the studied biodigester, it seems as the importance of the acetoclastic pathway shifts with time towards the hydrogenotrophic. Presence of *Methanoculleus* has been correlated with high biogas yield, whereas *Methanosaeta* has been linked to low biogas output (Abendroth et al., 2015). Our results are congruent with the characteristics of the biogas plant under study and previous literature reports of similar biodigesters (Stolze et al., 2015; Wirth et al., 2012). The plant operates under dry fermentation conditions with a high content of total solids and treats bovine residues that are rich in ammonia and alkaline (Goberna et al., 2009; Nettmann et al., 2010; Weiland, 2010). High ammonia concentrations might inhibit susceptible methanogens, while alkaline substances help stabilize the reactor pH. All these conditions appeared to contribute to the predominance of *Methanoculleus*, which potentially forms biofilms over the treated substrates in close proximity to acetate‐oxidizing bacteria allowing an adequate syntrophic relationship between H_2_ producers and consumers (Abendroth et al., 2015; Weiland, 2010; Wirth et al., 2012; Zhao et al., 2013). This would ensure an optimum H_2_ balance and an efficient operation of the biogas‐producing microbial community.

Concerning the associate bovine residues, each feedstock possessed a characteristic methanogenic community. As expected, leachate (L) composition was similar to the biodigester but maintained equal proportions of the orders Methanomicrobiales and Methanosarcinales (Figure 3c), which changed in the reactor towards a dominance of Methanomicrobiales. The manure (M) exhibited a uniform community of the three main methanogen orders including the Methanomicrobiales represented by the genus *Methanoculleus* that dominated the biodigester (Figure 5). Also, it appears to contribute the uncultured or unclassified component of the biogas‐producing community as the DGGE analysis revealed their significance during peak biogas production (Figure 2, Band 4, 15, 18). Of all feedstocks, the oxidation lagoon (OL) was the most diverse and almost exclusively possessed the genera *Methanosaeta* (Methanosarcinales) and *Methanolinea* (Methanobacteriales), observed as relevant members in the digester. In accordance to the literature (Zhou et al., 2010), rumen (R) was almost entirely dominated by the order Methanobacteriales represented by 10 species of the genus *Methanobrevibacter*, the most abundant being *M. ruminantium*. However, these methanogens did not proliferate in the biodigester, neither the second most abundant species in this community, *M. mazei*. This species was also a major component of manure (M), probably associated to the diet of the animals. Overall, feedstocks that significantly contribute to the biogas‐producing microbial community in the biodigester were leachate (L), as expected, and oxidation lagoon (OL) and manure (M) more than rumen (R).

Finally, during anaerobic digestion of the bovine residues three distinct microbial community profiles of the bacterial and archaeal component were observed, with the exception of Eukarya mainly represented by a stable presence of fungi. These changes appeared to correlate to each stage of biogas production. At the beginning of the run (samples B17 and B22), during the first 6 days, an adaptation phase was observed where no biogas was produced. This period was followed by biogas production, in the middle of the run (B27, B29), and, as a final phase, the last days of the run (B04) when biogas production reached zero.

## 5 CONCLUSION

At high taxonomic level, the two sets of information from DGGE and metagenomics correlated to some extent. Relevant methanogen genera as *Methanoculleus* and *Methanobrevibacter* were underestimated in the DGGE analysis. However, this technique was indispensable to discern the role that uncultured or unidentified methanogens played during biogas generation. The 10‐ton dry digester presented a diverse and dynamic community of bacteria and methanogens, which correlated to a particular stage during biogas production. These community profiles appeared to be supported by specific members that characterized each feedstock or residue. Water from the oxidation lagoon and manure were the most relevant substrates, while rumen methanogenic members did not proliferate in the reactor. It was confirmed that leachate, as the biodigester microbial inoculum, adequately preserved the biogas‐producing microbial community. Therefore, we were able to correlate presence of certain microorganisms in the biodigester to type of feedstock, which could lead to bioaugmentation strategies by incorporating a higher proportion or an enriched microbial inoculum from the most relevant feedstocks. Process adjustments would help reduce the adaptation phase in the digester and, consequently, decrease retention time and increase biogas yield if augmented microorganisms could further breakdown the organic waste.

## CONFLICT OF INTERESTS

The authors declare no conflict of interest.

## AUTHOR CONTRIBUTIONS

CSG designed, analyzed and wrote metagenomic analysis, FACC performed DGGE experiment, JFRL, BTP and JHSC provided the biological material, designed the study and revised the manuscript, and AP designed the study, analyzed data and wrote the manuscript. All authors read and approved the final manuscript.

## ETHICS STATEMENT

None required.

## Data Availability

All data are included in the manuscript. DGGE sequences were deposited in the National Center for Biotechnology Information (NCBI) under accession numbers MH393448–MH393458 and metagenomic database as BioProject PRJNA378243. Additional data is provided in Appendix A, Supplementary Material.
