# Supplementary material for: Biogas‐producing microbial composition of an anaerobic digester and associated bovine residues
Source: Microbiologyopen. 2019 May 25;8(9):e00854. doi: 10.1002/mbo3.854 (PMC6741126; doi:10.1002/mbo3.854)
Supplement: Supplementary file 1 [file MBO3-8-e00854-s001.docx]

**APPENDIX A**

**TABLE S1** Phylogenetic assignment of sequenced DGGE bands from archaeal PCR products (accessed 25 March 2019)

| **Sample** | **NCBI** | |  | **MiDAS** | | | |
| --- | --- | --- | --- | --- | --- | --- | --- |
|  | **Closest sequence** (accession number; sample origin) | **Compared bases (%id)** |  | **Closest sequence**  (accession number; sample origin)  *representative microorganism in bold^1^ | **Abbre- viation** | **Order (Family)** | **Habitat** |
| Oxidation Lagoon (OL)-Band 1 | Uncultured Methanobrevibacter sp. clone D_D11 (AY454734; brackish estuary, Brazil) | 148/148  (99%) |  | Uncultured archaeon clone Luo-56 (HM573448; pig feces, China), **Methanobrevibacter ruminantium** **CR6^2^** (MH709100; rumen fluid cow, India) | Mbr | Methanobacteriales (Methanobacteriaceae) | Major player in bovine (Whitford et al. 2001) and dairy cattle rumen (King et al. 2011). |
| Oxidation Lagoon (OL)-Band 2 | Uncultured archaeon isolate DGGE gel band 29 (JX887881; gut wild pig, China) | 149/149 (100%) |  | Uncultured archaeon clone AARC85 (JQ245063; human feces African American, USA), **Methanobrevibacter smithii DSM2375** (ABYW01000015; human feces, Germany) | Mbs | Methanobacteriales (Methanobacteriaceae) | Major player together with M.gottschalkii in dairy cattle rumen (King et al. 2011). |
| Oxidation Lagoon (OL)-Band 3 | Uncultured archaeon clone arch8 (GQ369773; solid municipal wastes, UK) | 139/139 (100%) |  | Uncultured archaeon clone A1-lcfa (HF955499; anaerobic LCFA-degrading enrichment cultures, Portugal), **Methanospirillum hungatei JF-1 DSM864** (JQ346747; culture collection) | Msh | Methanomicrobiales (Methanospirillaceae) | Isolated from sewage sludge (Ferry et al. 1974). |
| Leachate (L)-Band 1 | Uncultured Methanobrevibacter sp. clone D_D11 (AY454734; brackish estuary, Brazil) | 143/143 (100%) |  | Uncultured archaeon clone RI-RM-N091 (JF500590; nilgai/blue bull feces, India), **Methanobrevibacter ruminantium** **CR6^1^** (MH709100; rumen fluid cow, India) | Mbr | Methanobacteriales (Methanobacteriaceae) | Major player in bovine (Whitford et al. 2001) and dairy cattle rumen (King et al. 2011). |
| Leachate (L)-Band 2 | Uncultured Methanobrevibacter sp. clone DI_B05 (AY454733; brackish estuary, Brazil) | 139/141  (99%) |  | Uncultured bacterium clone APC-3439-J3B2 (KF616725; deep see Hydrate Ridge, USA) | UMb | Methanobacteriales (Methanobacteriaceae) | Brackish estuary, Brazil. |
| Leachate (L) – Band 3 | Uncultured archaeon clone SB-BJ21 (EF639564; Valley of Lake Pontchartrain, IL, USA) | 148/148 (100%) |  | Uncultured archaeon clone SYNH02_C3-03A-092 (JQ245664; mud volcano, Taiwan) | Uc1 | NA | Valley of Lake Pontchartrain, IL, USA. |
| Rumen (R) – Band1 | Uncultured archaeon clone Luo-55 (HM573447; pig manure, China) | 147/148  (99%) |  | Uncultured archaeon clone Luo-41 (HM573433; pig feces, China), **Methanosphaera stadtmanae** **DSM3091** (AY196684; human feces) | Mss | Methanobacteriales (Methanobacteriaceae) | Major player in bovine rumen (Whitford et al. 2001), represents 2% in dairy cattle rumen (King et al. 2011). |
| Rumen (R) – Band 2 | Uncultured Methanobrevibacter sp. isolate OTU_265 (LT624906; anaerobic digestion plant, Belgium) | 148/148 (100%) |  | Uncultured euryarchaeote clone 5 (DQ985542, goat rumen), **Methanobrevibacter boviskoreani JH1** (BAGX02000040; rumen native cattle, Korea) | Mbb | Methanobacteriales (Methanobacteriaceae) | Novel methanogen species from rumen of native Korean cattle (Lee et al. 2013). |
| Rumen (R) – Band 3 | Uncultured Methanobrevibacter sp. isolate OTU_289 (LT624911; anaerobic digestion plant, Belgium) | 145/146  (99%) |  | Uncultured euryarchaeote clone 9 (HM998286; swine manure), **Methanobrevibacter boviskoreani JH1** (BAGX02000040; rumen native cattle, Korea) | Mbb | Methanobacteriales (Methanobacteriaceae) | Novel methanogen species from rumen of native Korean cattle (Lee et al. 2013). |
| Rumen (R) – Band 4 | Uncultured archaeon isolate DGGE gel band 29 (JX887881; gut gilt pig, China) | 147/149  (99%) |  | Uncultured archaeon clone AARC85 (JQ245063; human feces African American, USA), **Methanobrevibacter smithii DSM2375** (ABYW01000015; human feces, Germany) | Mbs | Methanobacteriales (Methanobacteriaceae) | Major player together with M. gottschalkii in dairy cattle rumen (King et al. 2011). |
| Manure (M) – Band 1 | Uncultured Methanobrevibacter sp. ARC_OTU_35 (LT844496; anaerobic digestion plant, Belgium) | 146/146 (100%) |  | Uncultured archaeon clone RI-RM-N042 (JF500634, nilgai/blue bull feces, India) | UMb2 | Methanobacteriales (Methanobacteriaceae) | Anaerobic digestion plant, Belgium. |
| Manure (M) – Band 2 | Uncultured archaeon isolate DGGE gel band 29 (JX887881; gut gilt pig, China) | 151/151 (100%) |  | Uncultured archaeon clone AARC85 (JQ245063; human feces African American, USA), **Methanobrevibacter smithii DSM2375** (ABYW01000015; human feces, Germany) | Mbs | Methanobacteriales (Methanobacteriaceae) | Dominant archeon in human gut ecosystem, 10% of anaerobes in colon of healthy adults (Samuel et al. 2007). |
| Manure (M) – Band 3 | Uncultured Methanosarcina sp. clone S1b2 (MH122758; methane based biofilm batch reactor, China) | 147/147 (100%) |  | Uncultured archaeon clone AARC78 (JQ245045; human feces African American, USA), **Methanosarcina mazei C16** (AY196685; digester sludge, USA) | Msm | Methanosarcinales  (Methanosarcinaceae) | Isolated from sludge in a laboratory digester (35 ºC) fed on urban waste, domestic sludge and animal wastes (Mah, 1980). |
| Manure (M) – Band 4 | Uncultured Methanoculleus sp. isolate DGGE gel band 13B (KF358388; Luzhou-flavor pit mud, china) | 147/147 (100%) |  | Uncultured archaeon clone LR-13 (DQ302471; soda lake water, India), **Methanoculleus marisnigri JR1** (CP000562; anoxic sediments of the Black Sea) | **Mcm** | Methanomicrobiales (Methanomicrobiaceae) | Isolated from anoxic sediments of the Black Sea (Anderson et al. 2009). |
| Biodigester run: |  |  |  |  |  |  |  |
| B17 (17.01.2014) – Band 1 | Uncultured archaeon clone SB-BJ21(EF639564; Valley of Lake Pontchartrain, USA) | 150/150 (100%) |  | Uncultured archaeon clone SYNH02_C3-03A-092 (JQ245664; mud volcano, Taiwan) | Uc1 | NA | Valley of Lake Pontchartrain, USA. |
| B20 (20.01.2014) – Band 1 | Uncultured archaeon clone A0610D001_ P02 (AB655454; rice field, Japan) | 141/142  (99%) |  | Uncultured Crenarchaeotes archaeon (CU916834; mesophilic anaerobic digester treating municipal sludge, France) | Uc3 | NA | Rice field, Japan. |
| B20 (20.01.2014) – Band 2 | Uncultured archaeon clone NBLA310G (GU388935; anaerobic digester, USA) | 143/144  (99%) |  | Uncultured archaeon clone NBLA17H (GU388877; anaerobic digester treating food waste, USA), **Methanolinea mesophila TNR** (AB447467; rice field soil, Japan) | Mlm | Methanobacteriales (Methanoregulaceae) | Isolated from rice field soil (mesophilic), Taiwan (Sakai et al. 2012). |
| B27 (27.01.2014) – Band 1 | Uncultured archaeon clone ASP4 (JF980513; anaerobic digester, Canada) | 142/141  (99%) |  | Uncultured archaeon clone 4H7 (HQ678092; anaerobic digester treating swine manure, Canada), Candidatus Methanomethylophilus alvus Mx1201 (CP004049; human gut, France) | Uc2 | NA | Anaerobic digester, feathers and livestock waste, Canada (Xia et al. 2012). |
| B27 (27.01.2014) – Band 2 | Methanosaeta sp. clone A2281 (HQ133126; methagenomic consortium, China) | 139/142  (98%) |  | Uncultured archaeon clone Rpcp 5 (FJ347536; psychrophilic anaerobic granular biofilm treating contaminated water, Ireland), **Methanosaeta concilii GP-6** (CP002565, pear waste fermentor inoculated with sludge, Canada) | Msc | Methanosarcinales (Methanosaetaceae) | Isolated from a laboratory pear waste fermentor inoculated with anaerobic sludge (Patel & Sprott 1990). |
| B29 (29.01.2014) – Band 1 | Uncultured archaeon clone ASP4 (JF980513; anaerobic digester, Canada) | 140/142  (99%) |  | Uncultured archaeon clone 4H7 (HQ678092; anaerobic reactor treating swine manure, Canada), Candidatus Methanomethylophilus alvus Mx1201 (CP004049; human gut, France) | Uc2 | NA | Anaerobic digester, feathers and livestock waste, Canada (Xia et al. 2012). |
| B31 (31.01.2014) – Band 1 | Uncultured archaeon isolate DGGE gel band 29 (JX887881; gut gilt pig, China) | 149/149 (100%) |  | Uncultured archaeon clone AARC85 (JQ245063; human feces African American, USA), **Methanobrevibacter smithii DSM2375** (ABYW01000015; human feces, Germany) | Mbs | Methanobacteriales (Methanobacteriaceae) | Dominant archeon in human gut ecosystem, 10% of all anaerobes in colons of healthy adults (Samuel et al. 2007). |
| B31 (31.01.2014) – Band 2 | Uncultured archaeon isolate DGGE gel band 29 (JX887881; gut gilt pig, China) | 151/151 (100%) |  | Uncultured archaeon clone AARC85 (JQ245063; human feces African American, USA), **Methanobrevibacter smithii DSM2375** (ABYW01000015; human feces, Germany) | Mbs | Methanobacteriales (Methanobacteriaceae) | Dominant archeon in human gut ecosystem, 10% of all anaerobes in colons of healthy adults (Samuel et al. 2007). |
| B04 (04.02.2014) – Band 1 | Uncultured Methanoculleus sp. ARC_OTU_203 (LT844499; anaerobic digestion plant, Belgium) | 138/138  (99%) |  | Uncultured archaeon clone LR-13 (DQ302471; soda lake, India), **Methanoculleus marisnigri JR1** (CP000562; anoxic sediments of the Black Sea) | Mcm | Methanomicrobiales (Methanomicrobiaceae) | Isolated from anoxic sediments of the Black Sea (Anderson et al. 2009). |
| B04-R2 (04.02.2014 R2) – Band 1 | Uncultured archaeon clone ASP4 (JF980513; anaerobic digester, Canada) | 140/142  (99%) |  | Uncultured archaeon clone 4H7 (HQ678092; anaerobic reactor treating swine manure, Canada), Candidatus Methanomethylophilus alvus Mx1201 (CP004049; human gut, France) | Uc2 | NA | Anaerobic digester, feathers and livestock waste, Canada (Xia et al. 2012). |
| B04-R2 (04.02.2014 R2) – Band 2 | Uncultured archaeon clone ASP4 (JF980513; anaerobic digester, Canada) | 147/149  (99%) |  | Uncultured archaeon clone 4H7 (HQ678092; anaerobic reactor treating swine manure, Canada), Candidatus Methanomethylophilus alvus Mx1201 (CP004049; human gut, France) | Uc2 | NA | Anaerobic digester, feathers and livestock waste, Canada (Xia et al. 2012). |
| B04-R2 (04.02.2014 R2) – Band 3 | Uncultured Methanoculleus sp. ARC_OTU_203 (LT844499;  anaerobic digestion plant, Belgium) | 138/138  (99%) |  | Uncultured archaeon clone LR-13 (DQ302471; soda lake, India), **Methanoculleus marisnigri JR1** (CP000562; anoxic sediments of the Black Sea) | Mcm | Methanomicrobiales (Methanomicrobiaceae) | Isolated from anoxic sediments of the Black Sea (Anderson et al. 2009). |

^1^Based on the first 100 hits. ^2^Assignation from NCBI. NA: Not applicable.

**REFERENCES**

Anderson, I.J., Sieprawska-Lupa, M., Lapidus, A., et al., 2009. Complete genome sequence of Methanoculleus marisnigri Romesser et al. 1981 type strain JR1. Stand. Genomic Sci. 1(2), 189–196. doi:10.4056/sigs.32535

Altschul, S.F., Gish, W., Miller, W., Myers, E.W., Lipman D.J., 1990. Basic local alignment search tool. J. Mol. Biol. 215(3), 403–410. <https://doi.org/10.1016/S0022-2836(05)80360-2>

DeSantis, T.Z., Hugenholtz, P., Larsen, N., Rojas, M., Brodie, E.L., Keller, K., Huber, T., Dalevi, D., Hu, P., Andersen, G.L., 2006. Greengenes, a chimera-checked 16S rRNA gene database and workbench compatible with ARB. Appl. Environ. Microbiol. 72(7), 5069-5072. http://doi.org/10.1128/AEM.03006-05

Ferry, J.G., Smith, P.H., Wolfe, R.S., 1974. *Methanospirillum*, a new genus of methanogenic bacteria, and characterization of *Methanospirillum hungatei* sp. nov., Int. J. Syst. Bacteriol*.* 2, 465–469. doi: [10.1099/00207713-24-4-465](https://dx.doi.org/10.1099/00207713-24-4-465)

King, E.E., Smith, R.P., St-Pierre, B., Wright, A.-D.G., 2011. Differences in the rumen methanogen populations of lactating Jersey and Holstein dairy cows under the same diet regimen. Appl. Environ. Microbiol. 77, 5682–5687. http://doi.org/10.1128/AEM.05130-11

Lee, J.H., Kumar, S., Lee, G.H., Chang, D.H., Rhee, M.S., Yoon, M.H., Kim, B.C., 2013. Methanobrevibacter boviskoreani sp. nov., isolated from the rumen of Korean native cattle. Int. J. Syst. Evol. Microbiol. 63, 4196–4201. <http://doi.org/10.1099/ijs.0.054056-0>

Mah, R.A., 1980. Isolation and characterization of Methanococcus mazei. Curr. Microbiol. 3: 321-326. <https://doi.org/10.1007/BF02601895>

Patel, G.B., Sprott, G.D., 1990. *Methanosaeta concilii* gen. nov., sp. nov. ("*Methanothrix concilii*") and *Methanosaeta thermoacetophila* nom. rev., comb. nov. Int. J. Syst. Bacteriol. 40, 79-82. doi:[10.1099/00207713-40-1-79](https://dx.doi.org/10.1099/00207713-40-1-79)

Sakai, S., Ehara, M., Tseng, I.-C., Yamaguchi, T., Bräuer, S.L., Cadillo-Quiroz, H., Zinder, S.H., Imachi, H., 2012. Methanolinea mesophila sp. nov., a hydrogenotrophic methanogen isolated from rice field soil, and proposal of the archaeal family Methanoregulaceae fam. nov. within the order Methanomicrobiales. Int. J. Syst. Evol. Microbiol. 62, 1389-1395. doi: 10.1099/ijs.0.035048-0

Samuel, B.S., Hansen, E.E., Manchester, J.K., Coutinho, P.M., Henrissat, B., Fulton, R., Latreille, P., Kim, K., Wilson, R.K., Gordon, J.I., 2007. Genomic and metabolic adaptations of Methanobrevibacter smithii to the human gut. PNAS. 104, 10643-10648. <http://doi.org/10.1073/pnas.0704189104>

Xia, Y., Massé, D.I., McAllister, T.A., Kong, Y., Seviour, R., Beaulieu, C., 2012. Identity and diversity of archaeal communities during anaerobic co-digestion of chicken feathers and other animal wastes. Bioresour. Technol. 110,111-119. <https://doi.org/10.1016/j.biortech.2012.01.107>
