# Supplementary material for: Biogas‐producing microbial composition of an anaerobic digester and associated bovine residues
Source: Microbiologyopen. 2019 May 25;8(9):e00854. doi: 10.1002/mbo3.854 (PMC6741126; doi:10.1002/mbo3.854)
Supplement: Supplementary file 2 [file MBO3-8-e00854-s002.docx]

TABLE **S1** Single reads passing quality filters for each sample in the study

| **Sample** | **Number of raw reads** | **Number of quality filtered reads** |
| --- | --- | --- |
| Leachate (L) | 2,905,354 (311.1 MB) | 2,835,826 (301.1 MB) |
| Manure (M) | 3,702,010 (392.8 MB) | 3,618,648 (379.9 MB) |
| Oxidation lagoon (OL) | 2,181,306 (221.1 MB) | 2,148,974 (216.2 MB) |
| Rumen (R) | 5,609,734 (590.6 MB) | 5,525,788 (577.7 MB) |
| Biodigester run: |  |  |
| B17 (17.01.2014) | 1,350,974 (166.2 MB) | 1,287,824 (155.5 MB) |
| B22 (22.01.2014) | 2,146,006 (329.2 MB) | 2,078,262 (271.2 MB) |
| B27 (27.01.2014) | 2,738,066 (329.2 MB) | 2,657,064 (315.0 MB) |
| B29 (29.01.2014) | 4,578,652 (529.1 MB) | 4,532,532 (519.6 MB) |
| B04 (04.02.2014) | 3,152,680 (354.3 MB) | 3,088,728 (343.9 MB) |
