# Supplementary material for: Biogas‐producing microbial composition of an anaerobic digester and associated bovine residues
Source: Microbiologyopen. 2019 May 25;8(9):e00854. doi: 10.1002/mbo3.854 (PMC6741126; doi:10.1002/mbo3.854)
Supplement: Supplementary file 3 [file MBO3-8-e00854-s003.docx]

**TABLE S2** Number of paired reads and read relative abundance in parenthesis (≥1 %) of methanogens at the species level and potential methanogenesis process

| **Order** | **Species** | **Methanogenesis process** | **L** | **M** | **OL** | **R** | **Biodigester** | | | | |
| --- | --- | --- | --- | --- | --- | --- | --- | --- | --- | --- | --- |
|  |  |  |  |  |  |  | **B17** | **B22** | **B27** | **B29** | **B04** |
| Methanobacteriales | Methanobrevibacter boviskoreani | Hydrogenotrophic | -^a^ | - | - | 521  (3.1) | ND^b^ | - | ND | - | - |
|  | Methanobrevibacter millerae | Hydrogenotrophic | - | 121  (2.6) | 148  (1.4) | 951  (5.6) | - | - | - | - | - |
|  | Methanobrevibacter olleyae | Hydrogenotrophic | - | 71  (1.5) | - | 1061  (6.3) | - | - | - | - | - |
|  | Methanobrevibacter oralis | Hydrogenotrophic | - | - | - | 228  (1.3) | - | - | - | - | - |
|  | Methanobrevibacter ruminantium | Hydrogenotrophic | - | 123  (2.6) | - | 1,500  (8.9) | - | - | - | - | - |
|  | Methanobrevibacter smithii | Hydrogenotrophic | - | - | - | 219  (1.3) | - | - | - | - | - |
|  | Methanobrevibacter sp. A27 | Hydrogenotrophic | - | 58  (1.3) | - | 371  (2.2) | - | - | - | - | - |
|  | Methanobrevibacter sp. AbM4 | Hydrogenotrophic | - | - | - | 327  (1.9) | ND | - | - | - | ND |
|  | Methanobrevibacter sp. YE315 | Hydrogenotrophic | - | 125  (2.7) | - | 638  (3.8) | - | - | - | - | - |
|  | Methanobrevibacter wolinii | Hydrogenotrophic | - | - | - | 355  (2.1) | - | - | - | - | - |
|  | Methanolinea sp. SDB | Hydrogenotrophic | 1,345  (1.5) | - | 584  (5.6) | - | 3,102  (9.7) | 8,539  (12.6) | 3,163  (8.4) | 12,116  (13.8) | 1,727  (3.4) |
|  | Methanolinea tarda | Hydrogenotrophic | 1,076  (1.2) | - | 1,180  (11.3) | - | 2,095  (6.6) | 6,853  (10.1) | 1,457  (3.9) | 4,237  (4.8) | 726  (1.4) |
|  | Methanosphaera sp. WGK6 | Methylotrophic | - | - | - | 266  (1.6) | - | - | - | - | - |
| Methanomicrobiales | Methanocorpusculum bavaricum | Hydrogenotrophic | - | 88  (1.9) | - | - | - | - | - | - | - |
|  | Methanoculleus bourgensis | Hydrogenotrophic | 4,008  (4.4) | - | - | - | 992  (3.1) | 818  (1.2) | 1,010  (2.7) | 2,207  (2.5) | 2,502  (4.9) |
|  | Methanoculleus chikugoensis | Hydrogenotrophic | - | 103  (2.2) | ND | ND | ND | ND | ND | ND | ND |
|  | Methanoculleus horonobensis | Hydrogenotrophic | 1,620  (1.8) | ND | - | - | - | - | 1,647  (4.4) | 2,888  (3.3) | 3,199  (6.3) |
|  | Methanoculleus marisnigri | Hydrogenotrophic | 2,476  (2.7) | 132  (2.8) | - | - | 595  (1.9) | 744  (1.1) | 1,990  (5.3) | 3,668  (4.2) | 3,725  (7.3) |
|  | Methanoculleus sediminis | Hydrogenotrophic | 1,472  (1.6) | 106  (2.3) | - | - | 317  (1.0) | - | 1,330  (3.5) | 2,265  (2.6) | 2,452  (4.8) |
|  | Methanoculleus sp. MAB1 | Hydrogenotrophic | 2,136  (2.3) | - | - | - | 624  (2.0) | - | 518  (1.4) | 1,132  (1.3) | 1,062  (2.1) |
|  | Methanoculleus sp. MH98A | Hydrogenotrophic | 1,214  (1.3) | 147  (3.2) | - | - | - | - | 1,361  (3.6) | 2,454  (2.8) | 3,036  (6.0) |
|  | Methanoculleus thermophilus | Hydrogenotrophic | 3,203  (3.5) | 50  (1.1) | - | - | 714  (2.2) | 721  (1.1) | 1,440  (3.8) | 2,435  (2.8) | 2,366  (4.6) |
|  | Methanofollis ethanolicus | Hydrogenotrophic | - | - | - | - | - | - | - | - | 1,060  (2.1) |
|  | Methanofollis liminatans | Hydrogenotrophic | - | - | - | - | - | - | - | - | 757  (1.5) |
|  | Methanoregula boonei | Hydrogenotrophic | - | - | 162  (1.6) | - | 329  (1.0) | 882  (1.3) | - | - | - |
|  | Methanoregula formicica | Hydrogenotrophic | - | - | 504  (4.8) | - | 792  (2.5) | 2,330  (3.4) | 479  (1.3) | 1,096  (1.3) | - |
|  | Methanosphaerula palustris | Hydrogenotrophic | - | - | 113  (1.1) | - | - | - | - | - | - |
|  | Methanospirillum hungatei | Hydrogenotrophic | - | - | 1,354  (13.0) | - | 516  (1.6) | 2,322  (3.4) | - | - | - |
| Methanosarcinales | Methanosaeta concilii | Acetotrophic | 4,152  (4.6) | - | 1,818  (17.4) | - | 4,291  (13.4) | 13,292  (19.6) | 5,443  (14.5) | 14,541  (16.6) | 1,971  (3.9) |
|  | Methanosaeta harundinacea | Acetotrophic | 17,880  (19.6) | - | 103  (1.0) | - | 3,372  (10.6) | 5,121  (7.5) | 1,249  (3.3) | 2,069  (2.4) | 846  (1.7) |
|  | Methanosarcina flavescens | Acetotrophic/ Hydrogenotrophic | 1,620  (1.8) | - | ND | - | 321  (1.0) | - | - | - | - |
|  | Methanosarcina mazei | Acetotrophic/ Hydrogenotrophic | 1,702  (1.9) | 500  (10.8) | 99  (1.0) | 1,990  (11.8) | - | - | - | - | 654  (1.3) |
|  | Methanosarcina sp. 1.H.T.1A.1 | Acetotrophic/ Hydrogenotrophic | - | 180  (3.9) | - | - | - | - | - | - | - |
| Unclassified | | | 41,918  (45.9) | 2,194  (47.2) | 2,379  (22.8) | 6,942  (41.0) | 10,221  (32.0) | 16,788  (24.7) | 12,684  (33.8) | 26,789  (30.6) | 21,903  (42.9) |
| <1% RA | | | 5,504  (6.0) | 654  (14.1) | 1,992  (19.1) | 1,581  (9.3) | 3,686  (11.5) | 9,494  (14.0) | 3,802  (10.1) | 9,640  (11.0) | 3,060  (6.0) |
| Total reads | | | 91,326 | 4,652 | 10,436 | 16,950 | 31,967 | 67,904 | 37,573 | 87,537 | 51,046 |

^a^Relative abundance <1%, considered in category <1% RA. ^b^ND = Not detected. Samples: L = leachate; M = manure; OL = oxidation lagoon; R = rumen; B = biodigester time series (B17 = 17.01.2014, B22 = 22.01.2014, B27 = 27.01.2014, B29 = 29.01.2014, B04 = 04.02.2014).
